# An Ab Initio Study of Pressure-Induced Changes of Magnetism in Austenitic Stoichiometric Ni_2_MnSn

**DOI:** 10.3390/ma14030523

**Published:** 2021-01-22

**Authors:** Martin Friák, Martina Mazalová, Ilja Turek, Adéla Zemanová, Jiří Kaštil, Jiří Kamarád, Martin Míšek, Zdeněk Arnold, Oldřich Schneeweiss, Monika Všianská, Martin Zelený, Aleš Kroupa, Jana Pavlů, Mojmír Šob

**Affiliations:** 1 Institute of Physics of Materials, v.v.i., Czech Academy of Sciences, Žižkova 22, CZ-616 62 Brno, Czech Republic; 394206@mail.muni.cz (M.M.); turek@ipm.cz (I.T.); zemanova@ipm.cz (A.Z.); schneew@ipm.cz (O.S.); 230038@mail.muni.cz (M.V.); kroupa@ipm.cz (A.K.); mojmir@ipm.cz (M.Š.); 2 Department of Chemistry, Faculty of Science, Masaryk University, Kotlářská 2, CZ-611 37 Brno, Czech Republic; houserova@chemi.muni.cz; 3 Institute of Physics, v.v.i., Czech Academy of Sciences, Na Slovance 2, CZ-182 21 Prague 8, Czech Republic; kastil@fzu.cz (J.K.); kamarad@fzu.cz (J.K.); misek@fzu.cz (M.M.); arnold@fzu.cz (Z.A.); 4 Faculty of Mathematics and Physics, Charles University, Ke Karlovu 5, CZ-121 16 Prague 2, Czech Republic; zeleny@ipm.cz; 5 Faculty of Mechanical Engineering, Institute of Materials Science and Engineering, Brno University of Technology, Technická 2896/2, CZ-616 69 Brno, Czech Republic; 6 Central European Institute of Technology, CEITEC MU, Masaryk University, Kamenice 5, CZ-625 00 Brno, Czech Republic

**Keywords:** Ni-Mn-Sn, alloys, pressure, magnetism, ab initio, stability, point defects, swaps

## Abstract

We have performed a quantum-mechanical study of a series of stoichiometric Ni${}_{2}$MnSn structures focusing on pressure-induced changes in their magnetic properties. Motivated by the facts that (i) our calculations give the total magnetic moment of the defect-free stoichiometric Ni${}_{2}$MnSn higher than our experimental value by 12.8% and (ii) the magnetic state is predicted to be more sensitive to hydrostatic pressures than seen in our measurements, our study focused on the role of point defects, in particular Mn-Ni, Mn-Sn and Ni-Sn swaps in the stoichiometric Ni${}_{2}$MnSn. For most defect types we also compared states with both ferromagnetic (FM) and anti-ferromagnetic (AFM) coupling between (i) the swapped Mn atoms and (ii) those on the Mn sublattice. Our calculations show that the swapped Mn atoms can lead to magnetic moments nearly twice smaller than those in the defect-free Ni${}_{2}$MnSn. Further, the defect-containing states exhibit pressure-induced changes up to three times larger but also smaller than those in the defect-free Ni${}_{2}$MnSn. Importantly, we find both qualitative and quantitative differences in the pressure-induced changes of magnetic moments of individual atoms even for the same global magnetic state. Lastly, despite of the fact that the FM-coupled and AFM-coupled states have often very similar formation energies (the differences only amount to a few meV per atom), their structural and magnetic properties can be very different.

## 1. Introduction

The Heusler alloys are one of the most prominent family of compounds currently studied due the presence of an extraordinary magneto-structural transition in their phase diagrams because this non-diffusive martensitic transition is accompanied nearly always by very pronounced changes of physical properties of the alloys [1]. Namely, nonstoichiometric Ni${}_{2}$MnX (X = Ga, In, Sn) compounds exhibit unusual magnetic behaviour originating from competing antiferromagnetic and ferromagnetic interactions between and inside different magnetic sub-lattices containing Mn atoms [1,2,3,4,5]. The stoichiometric austenite Ni${}_{2}$Mn-based alloys crystallize in the cubic Fm-3m structure (L2${}_{1}$, full Heusler) and exhibit a long range ferromagnetic arrangement of moments below the Curie temperature, see, e.g., Ref. [6] and references therein.

In contrast, the Ni-Mn-X compounds undergo several magnetic and magneto-structural transition connected with pronounced changes of physical properties [7,8,9,10,11,12,13,14]. Importantly, some of these interesting phenomena are not fully understood yet and their explanation can potentially result in a deeper exploitation of the multifunctional properties of these materials. Therefore, Ni-Mn-Sn materials have been intensively studied both experimentally and theoretically in previous years. Experimental methods include investigations of their structural and magnetic properties, such as Refs. [6,15,16] using, e.g., Mössbauer spectroscopy [4,17,18,19,20].

Theoretical calculations include earlier quantum-mechanical studies, e.g., Refs. [5,21,22,23,24,25,26] as well as phenomenological thermodynamic assessments by the CALPHAD method [27,28]. Regarding the latter, a thermodynamic description of the corresponding binary systems exists in literature for Mn-Ni [29,30,31], Mn-Sn [30,32,33] and the Ni-Sn [34,35]. However, there is very little thermodynamic information related to the Mn-Ni-Sn ternary system [36,37]. As quantum-mechanical calculations can provide necessary parameters related to the lattice stabilities of metastable or unstable phases (as outlined first in 2001 in papers [38,39,40,41]), we hope that our results can serve to this purpose, too.

Our *ab initio* calculations were partly inspired by a series of recent ab initio studies. These include, for example, the work by Dutta, Opahle and Hickel [42] who calculated and analyzed interface effects on the magnetic properties of layered Ni${}_{2}$MnGa/Ni${}_{2}$MnSn nanocomposite alloys. The authors found (i) the Ni spin moments at the interface changed by about 30% compared to the bulk value and (ii) the magneto-crystalline anisotropy of the multilayer systems may be understood by the additive contributions of the respective strained bulk phases. There is also an excellent review by Entel et al. [43] who discussed magnetic exchange interactions and stability of different magnetic states, ordering/disordering energies and martensite transformation in Heusler Ni-Mn-X (X = Ga, In, and Sn). Further, a comprehensive review of coupling phenomena in magnetocaloric materials was published recently by Waske et al. [44]. As strong coupling effects in magnetocaloric materials are the key factor to achieve a large magnetic entropy change, Waske et al. compiled results for atomic coupling, stress coupling, and magnetostatic coupling in a set of Heusler compounds including Ni${}_{2}$MnGa, Mn-rich Ni-Mn-Z (Z = Al, In, Sn, Sb) as well as other more complicated, e.g., quaternary materials. Regarding a materials design of new shape-memory materials, Zhang et al. [45] demonstrated that the substitution of Cu for Sn in Ni-Mn-Sn alloy enhanced the magnetic-field-induced reverse martensitic transformation. Next, Buchelnikov et al. [46] studied correlation effects in ground-state properties of Heusler alloys Ni-Mn-Ga and Ni-Mn-Ga-Sn comparing properties of ferro-/ferri- and antiferromagnetic phases. As another example, Benguerine et al. [47] compared structural, elastic, electronic, and magnetic properties of Ni${}_{2}$MnSb, Ni${}_{2}$MnSn and Ni${}_{2}$MnSb${}_{0.5}$Sn${}_{0.5}$ magnetic shape memory alloys. Other recent studies cover, for example, quite complicated materials, e.g., Ni-Co-Mn-Cr-Sn [48], Ni-Mn-(Sn,In) compounds [49], Ni-Co-Mn-In alloys [50], Ni-Co-Mn-Sn materials [51] or Ni${}_{40}$Co${}_{10}$Mn${}_{36}$Al${}_{14}$ alloys [52].

Our research was motivated by the fact that quantum-mechanical studies of pressure-induced changes in different magnetic states of stoichiometric Ni${}_{2}$MnSn with point defects, in particular swaps, are quite rare and, therefore, we aimed at filling this gap in common knowledge. We also have our own experimental data (discussed below) and we hope that our theoretical results can shed a new light on very intriguing pressure-induced changes in Ni-Mn-Sn compounds.

## 2. Methods

### 2.1. Quantum-Mechanical Calculations

Our ab initio calculations were performed using the Vienna Ab initio Simulation Package (VASP) [53,54] that implements the density functional theory [55,56]. We have employed projector augmented wave (PAW) pseudopotentials [57,58] (Ni_pv, Mn_pv and Sn_d versions from the potpaw_PBE.52 VASP database). The exchange and correlation energy was treated within the generalized gradient approximation (GGA) [59].

**Figure 1:**
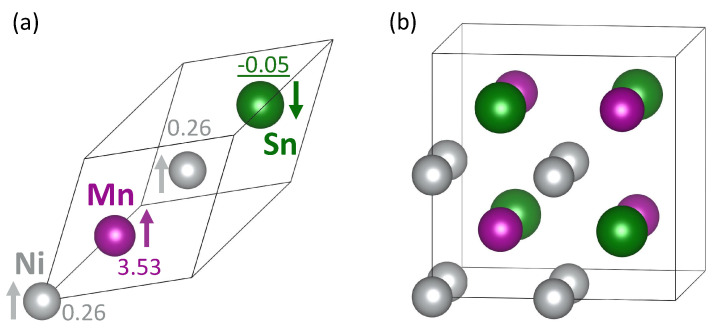
Schematic visualizations of 4-atom rhombohedral primitive unit cell (**a**) and 16-atom computational supercell (**b**) of the stoichiometric Ni${}_{2}$MnSn with the austenitic structure (L2${}_{1}$, so-called full Heusler structure). Arrows in part (**a**) indicate the orientation of local magnetic moments and numbers accompanying them are their magnitudes in Bohr magnetons with negative values indicating an antiparallel orientation (also underlined for the sake of clarity).

We have used 16-atom supercells with the L2${}_{1}$ austenitic phase of stoichiometric Ni${}_{2}$MnSn (see Figure 1b) and its substitutional variants for swap-containing states. The plane-wave energy cut-off was equal to 700 eV and the product of the number of Monkhorst–Pack k-points and the number of atoms was equal to 8192, i.e., 8 × 8 × 8 k-point mesh with its origin shifted to (1/2, 1/2, 1/2) in the case of 16-atom supercells. The Methfessel–Paxton order 1 smearing was applied with the smearing parameter equal to 0.23. All studied supercells were fully relaxed, i.e., their total energy was minimized with respect to their atomic positions, cell shape as well as the volume and the forces were reduced under 0.005 eV/Å. The calculations of either ferromagnetically and antiferromagnetically coupled magnetic moments of swapped atoms (discussed below) were initially started with the local magnetic moments of swapped atoms having either parallel or antiparallel orientation, respectively.

### 2.2. Experiments

The Czochralski method was used for preparation of single-crystal of Ni${}_{2}$MnSn compound. The method was implemented in a tri-arc furnace with water cooled copper crucible and with a tungsten rod as a seed. The starting polycrystalline material was melted and kept far above the melting temperature for one hour before the growing process was started. The melt was slowly cooled down to the vicinity of the melting temperature and the pulling rate was set from 10 to 15 mm/h. The Energy-Dispersive X-ray spectroscopy (EDX) was used to check the stoichiometric Ni${}_{2}$MnSn composition and the neutron Laue experiment confirmed formation of a single crystal of acceptable quality. The magnetic measurements at ambient and high pressure were performed on the MPMS-7T magnetometer (Quantum Design).

The high-pressure experiments were carried out in pressure range up to 1 GPa using the non-magnetic miniature Cu-Be pressure cell [60]. The magnetisation isotherms were measured in the temperature range from 5 K to 395 K in magnetic field up to 5 T. A decrease of saturated magnetization of the stoichiometric Ni${}_{2}$MnSn alloy under pressure is very weak and hence, it is superimposed by side effects resulting from pressure effect on magneto-crystalline anisotropy (Ha ≈ 500 Oe) and magnetic domain structure at low magnetic field range. Due to a highly time-consuming pressure experiments, limited experimental data were available only to determine the effect of pressure on magnetization. Unfortunately in this case, any fit cannot be done and presented. The presented values of experimental d(lnμ/d*p*) were determined as $1/\mu_{0}$(dμ/d*p*). To avoid unreliable values of d(ln$\mu_{0}$)/d*p*, the effect of pressure on saturated magnetization μ(5K,5T) of the alloy, d(lnμ(5K,5T))/d*p* = −(2.5± 0.2)·10${}^{-3}$ GPa${}^{-1}$, has been presented in our previous paper [6] suggesting that it is the most suitable magnitude for a description of the effect of pressure on the magnetic ground state of the alloy.

## 3. Results

Starting with the stoichiometric austenitic Ni${}_{2}$MnSn, our theoretical value of its lattice parameter, 6.059 Å, agrees very well with other previously published theoretical results (6.046 Å in Ref. [61] and 6.022 Å in Ref. [21]) as well as experimental value of 6.055 Å in Ref. [15]. The calculated values of local magnetic moments corresponding to its ground state are listed in Figure 1a. The magnetic moments obtained for Ni, Mn and Sn atoms, 0.26 $\mu_{B}$, 3.53 $\mu_{B}$ and $-0.05\;\mu_{B}$, respectively, are similar to previous theoretical values (0.21 $\mu_{B}$, 3.73 $\mu_{B}$ and $-0.05\;\mu_{B}$, respectively [21]) as well as experimental data of 3.60 to 3.75 $\mu_{B}$ reported for Mn in Ref. [62].

**Figure 2:**
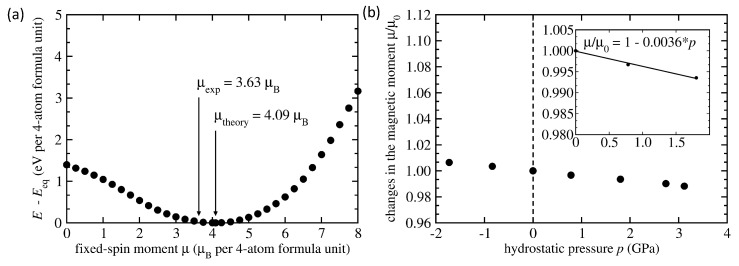
Computed total energy difference of the austenitic stoichiometric Ni${}_{2}$MnSn with different values of the fixed total magnetic moment with respect to the energy of the ground state (**a**). Arrows indicate experimental value of the magnetic moment and the theoretical value corresponding to the minimum of the energy. Part (**b**) shows calculated pressure-induced changes of the total magnetic moment μ relative to its zero-pressure value $\mu_{0}$. The inset contains a linear fit through the values in the lower-pressure region and its parameters including the value of d(lnμ)/d*p* close to the zero-pressure magnetic moment $\mu_{0}$ approximated as $\left(1/\mu_{0}\right)$ dμ/d*p* = d$(\mu /\mu_{0})/$d*p* = −0.0036 GPa${}^{-1}$.

The magnetic state in Figure 1a corresponds to the energy minimum on the dependence of the energy as a function of the total magnetic moment of the supercell shown in Figure 2. The visualized dependence was obtained when using so-called fixed spin moment (FSM) type of calculations. In the FSM calculations, the total magnetic moment is fixed to a specific value and the energy is minimized by changing all other degrees of freedom (atomic positions, local atomic magnetic moments, supercell shape and volume) with this constrained value of the total magnetic moment. We have not found any other stable magnetic state except for that one exhibiting the magnetic moment $\mu_{\mathrm{theory}}$ = 4.09 $\mu_{B}$ per 4-atom formula unit of Ni${}_{2}$MnSn. This value is higher than the reported experimental value of 3.63 $\mu_{B}$ per 4-atomic formula unit [6] and we will extensively discuss this discrepancy below.

We have also theoretically determined pressure-induced changes in the total magnetic moment and our results are shown in Figure 2b. Using the linear fitting function and its parameters (see Figure 2b), it is possible to determine the pressure derivative (of the logarithm) of the total magnetic moment d(lnμ)/d*p* = (1/μ) dμ/d*p* approximatively close to the zero-pressure value $\mu_{0}$ using the formula $\left(1/\mu_{0}\right)$ dμ/d*p* = d$(\mu /\mu_{0})/$d*p* = −0.0036 GPa${}^{-1}$. The computed value qualitatively agrees with experiments but the quantitative agreement is not so good as the experimental value reported in Ref. [6] is smaller, d(ln$\mu_{\exp})/$d*p* = −0.0025 GPa${}^{-1}$. Therefore, below we analyze the impact of different defects on the magnetic state and its pressure-induced changes in austenitic phases of Ni${}_{2}$MnSn. In particular, Sokolovskiy et al. in Ref. [61] showed the importance of (i) so-called structural defects (such as Mn atoms swapping with the Ni atoms in the same amount) and (ii) excess Mn atoms (on the expense of the Sn atoms on the Sn sublattice) in both stoichiometric and off-stoichiometric cases. Therefore, here we analyse the effect of different types of anti-site (swapped) atoms in the stoichiometric Ni${}_{2}$MnSn.

**Figure 3:**
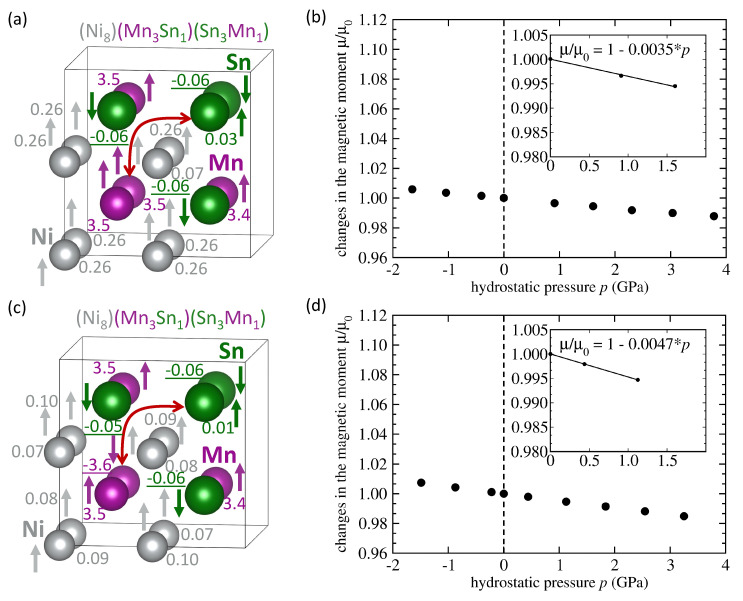
A 16-atom computational supercell of the austenitic stoichiometric Ni${}_{2}$MnSn with one Mn atoms swapping one Sn atom as indicated by the red arrow (**a**) with the local magnetic moment of the swapped Mn atom (on the Sn sublattice) having the orientation equal as that of Mn atoms at the Mn sublattice. Included are also values of local magnetic moments (in Bohr magnetons) corresponding to a minimum-energy zero-pressure state. Part (**b**) shows the calculated pressure-induced changes of the total magnetic moment of the state in part (**a**) together with the corresponding linear fit. Figure (**c**) shows a state with the swapped Mn atom having its local magnetic moments anti-parallel to the Mn atoms on the Mn sublattice. Part (**d**) shows calculated pressure-induced changes of the total magnetic moment of the state shown in part (**c**) together with the corresponding linear fit. Magnitudes of local magnetic moments are listed in Bohr magnetons and negative underlined values mean antiparallel orientation.

First, we have studied one Mn atom swapping with one Sn atom in our 16-atom supercell while preserving the overall stoichiometry of Ni${}_{2}$MnSn. The swap is schematically indicated by a red arrow in Figure 3a,c and we emphasize that the red arrow has only a schematic meaning, i.e., it does not show the actual diffusion process of the swapping. We have analyzed the pressure dependence of magnetic moment of two types of states. First, the Mn anti-site atom couples ferromagnetically (FM) with the Mn atoms on the Mn sublattice and they all have parallel orientation of their local magnetic moments, see Figure 3a,b. Second, the local magnetic moment of the Mn atom on the Sn sublattice couples in an anti-ferromagnetic way (AFM) and its orientation is anti-parallel to that of local magnetic moments of Mn atoms on the Mn sublattice, see Figure 3c,d. Figure 3a,c show how complicated the magnetic states are (local magnetic moments of Ni and Mn atoms having many different magnitudes). The FM-coupled case has the total magnetic moment (4.03 $\mu_{B}$ per 4-atom formula unit) more than twice higher than the AFM-coupled state (1.87 $\mu_{B}$ per 4-atom formula unit). Inversely, the sensitivity to the applied pressures is significantly higher in the AFM-coupled state, d$(\mu /\mu_{0})/$d*p* = −0.0047 GPa${}^{-1}$, than in the FM-coupled one with d$(\mu /\mu_{0})/$d*p* = −0.0035 GPa${}^{-1}$.

**Figure 4:**
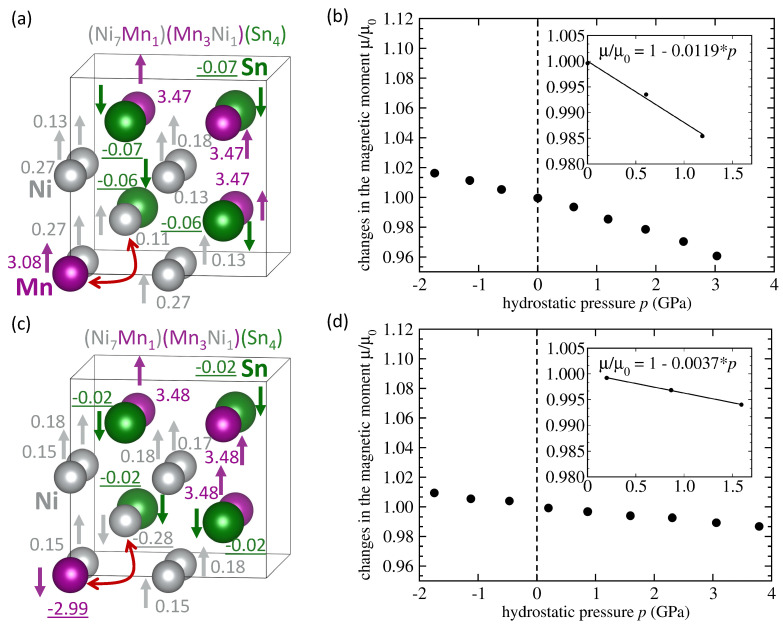
A schematic visualization of a 16-atom computational supercell of the austenitic stoichiometric Ni${}_{2}$MnSn (**a**) with one Mn atom swapping with one Ni atom (indicated by the red arrow) with the local magnetic moments of the swapped Mn atom having the orientation equal as that of the Mn atoms at the Mn sublattice. Part (**b**) shows calculated pressure-induced changes of the total magnetic moment of this state (the computed data points are accompanied by a linear fit and its parameters). Figure (**c**) shows a state with the swapped Mn atom having its local magnetic moments anti-parallel to those of Mn atoms on the Mn sublattice. Part (**d**) exhibits calculated pressure-induced changes of the total magnetic moment of the state shown in part (**c**) together with the corresponding linear fit. Magnitudes of local magnetic moments are listed in Bohr magnetons and negative underlined values mean antiparallel orientation.

Next, we analyze the impact of another type of swap when one Mn atom swaps with one Ni atom (per 16-atom computational cell), see Figure 4. Again, we have determined the minimum-energy state as well as its pressure-induced changes in the total magnetic moment for the swapped Mn atom on the Ni sublattice being coupled both ferromagnetically (Figure 4a,b) and anti-ferromagnetically (Figure 4c,d) to the Mn atoms on the Mn sublattice. The AFM-coupled state has significantly lower total magnetic moment (2.09 $\mu_{B}$ per 4-atom formula unit) than the FM-coupled one (3.75 $\mu_{B}$ per 4-atom formula unit) due to the fact that (i) the anti-site Mn atom on the Ni sublattice has opposite orientation of its magnetic moment, (ii) one Ni nearest neighbor of the swapped Mn atom has opposite orientation and (iii) some other Ni atoms have their local magnetic moments reduced, see Figure 4c. The sensitivity of the magnetic moment to the applied pressures of the FM-coupled state, d$(\mu /\mu_{0})/$d*p* = −0.0119 GPa${}^{-1}$, is more than three times higher than that of the AFM-coupled state, d$(\mu /\mu_{0})/$d*p* = −0.0037 GPa${}^{-1}$, see Figure 4b,d, respectively. It is worth noting that even the FM-coupled Mn atom on the Ni sublattice has its magnetic moment (3.08 $\mu_{B}$) lower than the Mn atom on the Mn sublattice (about 3.5 $\mu_{B}$).

**Figure 5:**
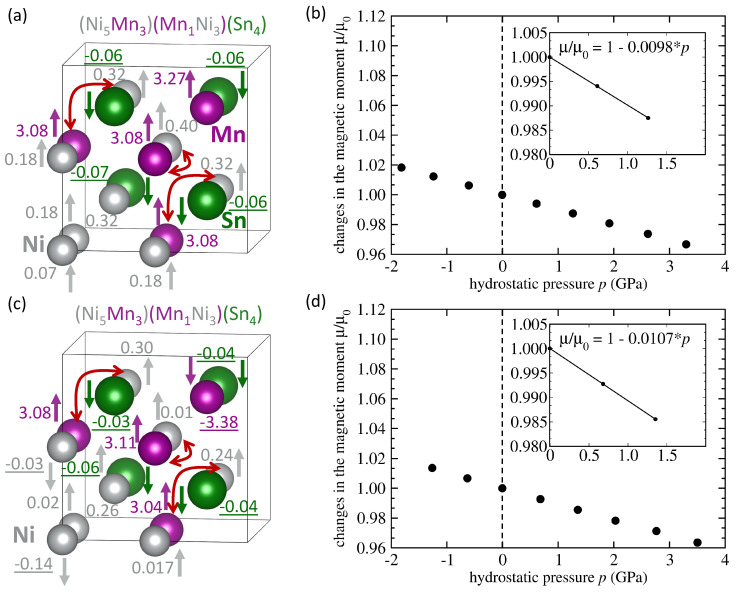
Similar set of figures as in Figure 4 but with three times more swapped atoms (three Mn atoms swapping with three Ni atoms, see red arrows). Schematic visualizations including local magnetic moments are shown in parts (**a**,**c**) for states FM and AFM coupled Mn atoms, respectively. The corresponding pressure-induced changes are presented in parts (**b**,**d**). Magnitudes of local magnetic moments are listed in Bohr magnetons and negative values indicate antiparallel orientation.

As the number of Mn-Ni swaps can be quite high (see Ref. [61]), we have also computed properties of states containing three such Mn atoms (per 16-atom supercell) swapping with Ni atoms, see Figure 5. As the ferromagnetically coupled Mn atoms on the Ni sublattice have their magnetic moments (3.08 $\mu_{B}$) again lower than the Mn atom on the Mn sublattice (3.27 $\mu_{B}$) the total magnetic moment of the FM-coupled state is lower (3.58 $\mu_{B}$ per 4-atom formula unit). Interestingly, the AFM-coupled state has the local magnetic moments of Mn atoms on the Ni sublattice still parallel to majority of Ni atoms but the single remaining Mn atom on the Mn sublattice flipped the orientation of its local magnetic moment into the anti-parallel one, see Figure 5c. The local magnetic moments of two Ni atoms on the Ni sublattice are flipped too, see Figure 5c. Consequently, the total magnetic moment of the AFM-coupled state, see Figure 5c, is much lower (1.57 $\mu_{B}$ per 4-atom formula unit) than that of the FM-coupled state (3.58 $\mu_{B}$ per 4-atom formula unit). The situation of the AFM-coupled state, when three Mn atoms have magnetic moments with the antiparallel orientation of one Mn atom on the Mn sublattice and their magnetic moments are decisive for the total magnetic moment of the whole supercell, is similar to that with only one Mn atom swapped, see Figure 4c, and the total magnetic moment (1.57 $\mu_{B}$ per 4-atom formula unit) is quite close to that with only one Mn swap in Figure 4d, that was equal to 2.09 $\mu_{B}$ per 4-atom formula unit. As far as the sensitivities to the pressure are concerned, they are quite high for both the FM-coupled (d$(\mu /\mu_{0})/$d*p* = −0.0098 GPa${}^{-1}$) and AFM-coupled state (−0.0107 GPa${}^{-1}$).

**Figure 6:**
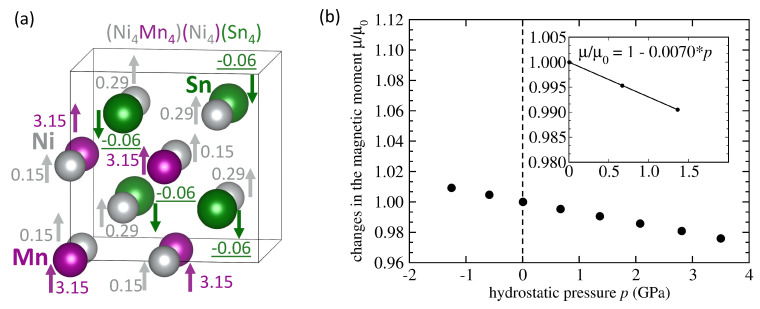
A 16-atom computational supercell of the stoichiometric Ni${}_{2}$MnSn with the inverse Heusler structure (**a**) and local magnetic moments corresponding to a minimum-energy zero-pressure state. Part (**b**) shows calculated pressure-induced changes of the total magnetic moment of the state shown in part (**a**) together with the corresponding linear fit.

If the number of Mn-Ni swaps would further grow to four, the structure becomes the inverse Heusler structure. The corresponding results are shown in Figure 6. We were able to find only one magnetic state when all Mn atoms are FM-coupled to Ni atoms, see in Figure 6a. The magnitude of magnetic moments of Mn atoms is lower (3.15 $\mu_{B}$) than in the full Heusler structure shown in Figure 1 (3.53 $\mu_{B}$) and, consequently, the total magnetic moment is also lower (3.51 $\mu_{B}$ per 4-atom formula unit). Its sensitivity to the pressure, d$(\mu /\mu_{0})/$d*p* = −0.0078 GPa${}^{-1}$, see Figure 6b, is about twice higher than in the case of the defect-free Ni${}_{2}$MnSn where d$(\mu /\mu_{0})/$d*p* = −0.0036 GPa${}^{-1}$.

**Figure 7:**
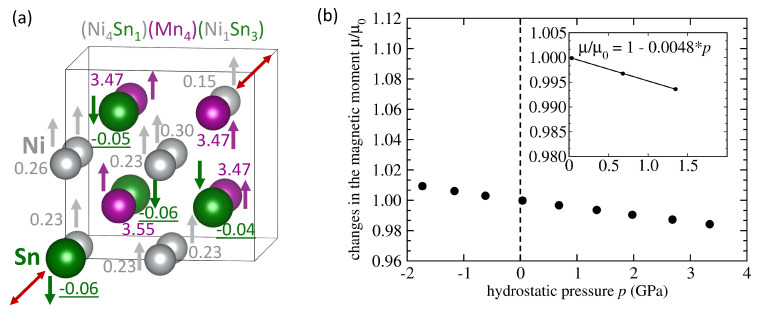
A schematic visualization of a 16-atom computational supercell of the austenitic stoichiometric Ni${}_{2}$MnSn with one Sn atoms swapping one Ni atom as indicated by the red arrows (**a**) accompanied by values of local magnetic moments (in Bohr magnetons) corresponding to a minimum-energy zero-pressure state. Part (**b**) shows calculated pressure-induced changes of the total magnetic moment of the state shown in part (**a**) together with the corresponding linear fit.

The next studied type of the swap is that of Ni and Sn atoms and our results are shown in Figure 7. Furthermore, in this case we were able to find only one state in which are Mn atoms FM-coupled to the Ni atoms. Due to the fact that all Mn atoms are located on their sublattice of the full Heusler structure, their local magnetic moments are 3.47 and 3.55 $\mu_{B}$ and the total magnetic moment is again higher, 4.04 $\mu_{B}$ per 4-atom formula unit, see Figure 7a and the sensitivity to the pressure is higher d$(\mu /\mu_{0})/$d*p* = −0.0048 GPa${}^{-1}$, see Figure 7b, than that of the defect-free Ni${}_{2}$MnSn, where d$(\mu /\mu_{0})/$d*p* = −0.0036 GPa${}^{-1}$.

## 4. Discussion

**Figure 8:**
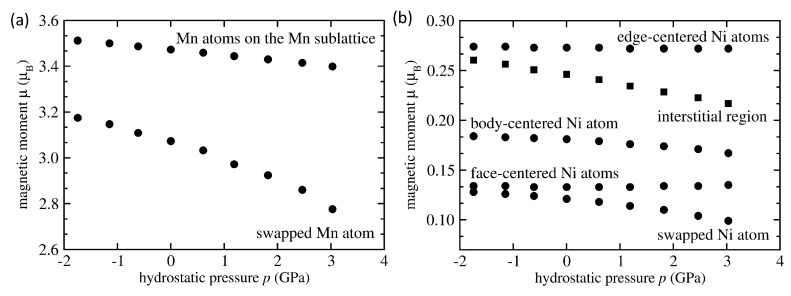
Computed changes of the magnetic moment of individual Mn atoms (**a**) as well as those of Ni atoms and in the interstitial region (**b**) in the case of FM-coupled single Mn-Ni swap.

The magnetism of Ni${}_{2}$MnSn is complicated due to a number of reasons. First, local magnetic moments of the three constituting elements span two orders of magnitude with Mn having magnetic moments as high as 3.7 $\mu_{B}$, Ni atoms exhibit quite a wide range between 0.1 and 0.7 $\mu_{B}$ and Sn atoms can have the magnitudes of their magnetic moments as low as 0.01 $\mu_{B}$. Second, pressure-induced changes are, in principle, different for each element and even for each individual atom of the same chemical species in the case when these atoms are in different atomic environments. Further, due to the existence of distinctly different magnetic states, such as high-spin vs. low-spin, the pressure-induced changes can then, in principle, lead to discontinuous changes of magnitudes of local atomic moments and, consequently, also abrupt changes in the total magnetic moment. Our study, nevertheless, predicts smooth nearly-linear dependencies of total magnetic moments (as shown in figures above), at least in the studied range of pressure, i.e., up to about 4 GPa. As the complexity can be hidden on the level of individual atoms, we have thoroughly analyzed local magnetic moments of atoms. The FM-coupled Mn-Ni-swapped states in Figure 4b were chosen as an example. We show their pressure-induced changes in Figure 8. The local magnetic moments of Mn atoms decrease with increasing pressure but the swapped Mn atom on the Ni sublattice (smaller values in Figure 8a) responds more sensitively than the Mn atoms on the Mn sublattice (higher values in Figure 8a, with three atoms having overlapping trends). The slope of the local magnetic moment of the swapped Mn atom is also different (higher) for positive pressures than for negative ones while the magnetic moments of Mn atoms on the Mn sublattice have the slope very similar for both pressure regions. The magnetic moments of Ni atoms in Figure 8b cover a broad range of values and also exhibit qualitatively different pressure dependencies. Those atoms with the highest magnitude of magnetic moment have them practically constant for different pressures, while the magnetic moments of Ni atoms with the lowest magnitude decreases steeply with increasing pressure and the decreasing trends are yet steeper for positive pressures (when compared with the negative ones).

Next, as we evaluate the local magnetic moments of atoms as the difference between the up and down electronic densities inside spheres around the atomic positions and these spheres do not overlap, there is also some electronic density in the interstitial region among the atoms and it is also spin-polarized. Its magnetic moment quite steeply decreases with increasing pressure as shown in Figure 8b. Our results also allow for evaluation of pressure sensitivity of local magnetic moments of individual atoms as well as that in the interstitial region. As far as the Mn atoms are concerned, the three with the magnetic moment of 3.47 $\mu_{B}$ have the pressure derivative d$(\mu /\mu_{0})/$d*p* = −0.0067 GPa${}^{-1}$ while that with the magnetic moment 3.08 $\mu_{B}$ has the sensitivity to the pressure about three times higher with d$(\mu /\mu_{0})/$d*p* = −0.0215 GPa${}^{-1}$.

Regarding the Ni atoms, the three of them with the magnetic moment equal to 0.27 $\mu_{B}$ and the three that have the magnetic moment equal to 0.13 $\mu_{B}$ are practically insensitive to the applied pressures. In contrast, the Ni atoms with the local magnetic moments of 0.18 $\mu_{B}$ and 0.11 $\mu_{B}$ are much more sensitive to applied pressures with d$(\mu /\mu_{0})/$d*p* equal to −0.0182 GPa${}^{-1}$ and −0.0409 GPa${}^{-1}$, respectively. The interstitial region with the magnetic moment equal to 0.24 $\mu_{B}$ is also very sensitive to the applied pressures with d$(\mu /\mu_{0})/$d*p* = −0.0361 GPa${}^{-1}$. Apparently, the pressure-induced changes in the total magnetic moment can represent rather complex interplay of changes at the level of individual atoms. However, it should be noted that some of the trends shown in Figure 8 are no longer linear and the coefficients mentioned above were determined from (i) the zero-pressure state and (ii) that with the lowest non-zero positive pressure. Consequently, an estimated error bar is about ±0.005 GPa${}^{-1}$ here.

Lastly, the local magnetic moments of the Sn atoms are negative (antiparallel) and indeed very small with their magnitudes comparable with (or even smaller than) an expected error-bar of our calculations (about 0.05 $\mu_{B}$). Therefore, we do not visualize them here. The pressure sensitivities of Sn atoms are extremely difficult to evaluate as the pressure-induced changes (of already very small magnitudes of individual Sn atoms) are practically zero. It should be also noted that the values of the local magnetic moments slightly depend on the radii of the spheres centered at atomic positions (here equal to 1.058 Å for Ni, 1.323 Å for Mn and 1.566 Å for Sn) where we evaluate electronic densities. In particular, the interstitial charge density is affected by pressure-induced volume reduction because the atomic spheres mentioned above keep their radii while the volume of the crystal is changed.

Another factor adding into the complexity of magnetism of Ni${}_{2}$MnSn is the fact that a change from the FM coupling to the AFM one often does not represent a significant energy difference and, consequently, energetically nearly degenerated and magnetically frustrated states can easily occur already at low temperatures. In order to evaluate this aspect more quantitatively, we have determined the formation energy $E_{f}$ of all studied systems in their lowest-energy zero-pressure state.

In particular, for all our atomic configurations with the stoichiometry ${\mathrm{Ni}}_{8}{\mathrm{Mn}}_{4}{\mathrm{Sn}}_{4}$ the formation energy was computed as $E_{f}=\left(E\left({\mathrm{Ni}}_{8}{\mathrm{Mn}}_{4}{\mathrm{Sn}}_{4}\right)-8\cdot E\left(\mathrm{Ni}\right)-4\cdot E\left(\mathrm{Mn}\right)-\right.$$\left.4\cdot E(\mathrm{Sn})\right)/16$, where $E({\mathrm{Ni}}_{8}{\mathrm{Mn}}_{4}{\mathrm{Sn}}_{4})$ is the energy of 16-atom supercell of Ni${}_{8}$Mn${}_{4}$Sn${}_{4}$ and the E(Ni), E(Mn) and E(Sn) are energies (per atom) of face-centered-cubic (fcc) ferromagnetic Ni, α-Mn and diamond-structure Sn (α-Sn), respectively. As far as the energy of α-Mn is concerned, we have computed the energy of antiferromagnetic body-centered tetragonal (bct) Mn (a tetragonally deformed fcc γ-Mn) and then added an energy offset as in Ref. [63]. The formation energies listed in Table 1 show that the FM-coupled states are energetically preferred over the AFM-coupled ones in most studied cases.

**Figure 9:**
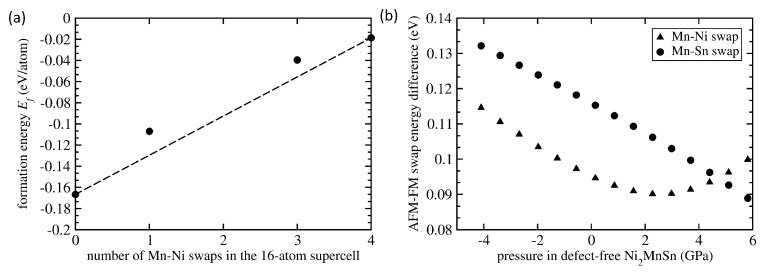
Computed compositional dependence of formation energy of systems with Mn-Ni swaps as a function of their number in 16-atom supercells modeling stoichiometric Ni${}_{2}$MnSn (**a**). Part (**b**) shows a pressure-dependence of the energy difference between AFM and FM-coupled states.

The AFM coupling is energetically preferred only in the stoichiometric Ni${}_{8}$Mn${}_{4}$Sn${}_{4}$ with three Mn atoms swapping with three Ni atoms but this systems is thermodynamically significantly destabilized by such a high concentration of swaps, i.e., the formation energy is significantly increased. An important aspect related to point defects is a concentration dependence of their properties. Focusing on Mn-Ni swaps, we have studied four different states with (i) zero defects (defect-free Ni${}_{8}$Mn${}_{4}$Sn${}_{4}$ with the L2${}_{1}$ full Heusler structure), (ii) one Mn-Ni swap, (iii) three Mn-Ni swaps and (iv) four Mn-Ni swaps (the Ni${}_{8}$Mn${}_{4}$Sn${}_{4}$ with the inverse Heusler structure). As the composition is equal in all states, we can directly compare their formation energies and the results are shown in Figure 9a.

If we connect the end points (the defects free Ni${}_{8}$Mn${}_{4}$Sn${}_{4}$ with the L2${}_{1}$ full Heusler structure and the the Ni${}_{8}$Mn${}_{4}$Sn${}_{4}$ with the inverse Heusler structure) then the states with one and three Mn-Ni swaps in the 16-atom supercells have energies above the line connecting the end members. This indicates that these defect-containing states are thermodynamically not stable with respect to the decomposition into these two phases (the full and inverse Heusler structures) and their number in experimental well-equilibrated samples would be low. On the other hand, our formation energies are those of static lattices without any entropy terms and we can speculate that, e.g., configuration entropy related to the Mn-Ni swaps can lower the free energy of these states under the tie line because the configuration entropy of both full and inverse Heusler structures is zero. The probability of occurrence of the states with Mn-Ni swaps would grow with temperature and they can be found in experimental samples.

Now, having all the above summarized results for swaps preserving the composition of Ni${}_{2}$MnSn, we can address the theory-experiment discrepancy related to the total magnetic moment and its pressure derivative. As the stoichiometic samples in Ref. [6] were cooled relatively slowly down to the room temperature, it is possible that (i) some Mn-Ni, Mn-Sn and Ni-Sn swaps were created at elevated temperatures but (ii) the system did not equilibrate during the slow cooling due to insufficient diffusion at lower temperatures (an experimentally detected concentration of vacancies is only 500 ppm [64]) and (iii) the defects stayed frozen in as regions (coherent inclusions) in the single-crystal lattice sharing the lattice parameter with the surrounding defect-free Ni${}_{2}$MnSn lattice. It is nevertheless difficult to estimate how numerous would the swaps be and how would the swapped Mn atoms couple to other Mn atoms.

A semi-quantitative estimate of the number of different types of swaps can be made on the basis of their defect formation energies. As all our systems have the same number of atoms, the swap formation energies of different swaps $E_{\mathrm{swap}-\mathrm{form}}$ can be evaluated from the energy of the swap-containing system $E_{\mathrm{swap}}$(Ni${}_{8}$Mn${}_{4}$Sn${}_{4}$) and the energy of the defect-free Ni${}_{8}$Mn${}_{4}$Sn${}_{4}$, i.e., $E_{\mathrm{defect}-\mathrm{free}}$(Ni${}_{8}$Mn${}_{4}$Sn${}_{4}$), as $E_{\mathrm{swap}-\mathrm{form}}=E_{\mathrm{swap}}\left({\mathrm{Ni}}_{8}{\mathrm{Mn}}_{4}{\mathrm{Sn}}_{4}\right)-E_{\mathrm{defect}-\mathrm{free}}\left({\mathrm{Ni}}_{8}{\mathrm{Mn}}_{4}{\mathrm{Sn}}_{4}\right).$ The swap formation energies of one Mn-Ni, Mn-Sn and Ni-Sn swap (in a 16-atom supercell) are 0.862 eV, 1.490 eV and 2.471 eV, respectively for FM-coupled states.

If we estimate the probability of occurrence of these swaps by the Boltzmann statistics, their numbers at a given temperature would exponentially decrease with increasing defect formation energy. All these FM-coupled swaps-containing states have their total magnetic moments lower (Mn-Ni: 3.75 $\mu_{B}$ per 4-atom formula unit, Mn-Sn: 4.03 $\mu_{B}$/f.u. and Sn-Ni: 4.04 $\mu_{B}$ per 4-atom formula unit) than that computed for defect-free Ni${}_{2}$MnSn (4.09 $\mu_{B}$ per 4-atom formula unit). In particular, it is true for the Ni-Mn swaps (3.75 $\mu_{B}$/f.u.) that we estimate to be the most populous. Therefore, the existence of these swaps in experimental samples would likely lower the total magnetic moment closer to the experimental value (3.63 $\mu_{B}$/f.u.). It should be, nevertheless, noted that the studied atomic configurations have been chosen so as to have a maximum symmetry, ideally a cubic one, while there is enormous number of other possible atomic configurations with swaps that can, in principle, exist in the stoichiometric Ni${}_{2}$MnSn lattice and in experimental samples.

Regarding the theory-experiment discrepancy in the pressure derivative of the logarithm of total magnetic moment of Ni${}_{2}$MnSn (theory: −0.0036 GPa${}^{-1}$ vs. experiment: −0.0025 GPa${}^{-1}$), it is difficult to find a simple explanation. Assuming the existence of the swaps discussed above in the form of coherent inclusions within the single-crystal Ni${}_{2}$MnSn lattice, small inclusions with swapped atoms will be elastically strained because they have equilibrium lattice parameters different from that of the defect-free Ni${}_{2}$MnSn (see Table 1). The applied hydrostatic pressure can further complicate their coherent co-existence and related elastic-deformation fields around them. The FM-coupled states with one Mn-Ni swap (per 16 atoms) has the equilibrium lattice parameter of 6.058 Å, i.e., nearly identical to that of the surrounding defect-free lattice (6.059 Å) and resulting strains will be small. However, the FM-coupled states with one Mn-Ni swap have pressure derivative of the logarithm of the magnetic moment (−0.0119 GPa${}^{-1}$) nearly three times bigger in magnitude than that of defect-free Ni${}_{2}$MnSn (−0.0036 GPa${}^{-1}$) and yet further away from the experimental value (−0.0025 GPa${}^{-1}$). The AFM-coupled Mn-Ni-swapped states have their equilibrium lattice parameter smaller (6.053 Å) and can become preferable for higher applied pressures. However, the energy of the AFM-coupled Mn-Ni swaps is higher than that of FM-coupled swaps (see Table 1) and the energy difference has even non-linear pressure dependence (see Figure 9b). The AFM-FM energy difference first decreases with increasing pressure, has a minimum for about 2.5 GPa and then increases again with increasing pressure. This non-linearity further complicates our analysis. Regarding the total magnetic moment of AFM-coupled Mn-Ni-swapped state (2.09 $\mu_{B}$ per 4-atom formula unit), it is about half of that of FM-coupled one ( 3.75 $\mu_{B}$/f.u.) and its pressure derivative of the logarithm of the magnetic moment (−0.0037 GPa${}^{-1}$) is lower (in its magnitude) than that of the FM-coupled states (−0.0119 GPa${}^{-1}$ discussed above) but still higher than that of the defect-free Ni${}_{2}$MnSn (−0.0036 GPa${}^{-1}$).

Considering the above mentioned coherency strains, the regions with Mn-Sn swaps would fit, from this perspective, better into the surrounding matrix if they are AFM-coupled because their equilibrium lattice parameter of 6.076 Å is closer to the equilibrium lattice parameter, 6.059 Å, of the defect-free Ni${}_{2}$MnSn than that of the FM-coupled state, 6.083 Å. However, the energy of AFM-coupled Mn-Sn swaps is higher than that of FM-coupled swaps (see Table 1) and the energy difference between them linearly decreases with increasing pressure in the surrounding matrix (see Figure 9b). The existence of these AFM-coupled Mn-Sn swapped states would further lower the overall magnetic moment of the whole system (matrix plus the regions with the Mn-Ni swaps) because the magnetic moment of AFM-coupled Mn-Sn swaps is only 1.87 $\mu_{B}$ per 4-atom formula unit, i.e., less than one half of the magnetic moment, 4.09 $\mu_{B}$/f.u., of defect-free 16-atom Ni${}_{2}$MnSn. However, the pressure derivative of the logarithm of the magnetic moment of the AFM-coupled Mn-Sn-swapped states (−0.0047 GPa${}^{-1}$) is higher (in magnitude) than that of the defect-free Ni${}_{2}$MnSn (−0.0036 GPa${}^{-1}$) while the FM-coupled Mn-Sn-swapped states have it slightly lower (−0.0035 GPa${}^{-1}$) than the defect-free Ni${}_{2}$MnSn (−0.0036 GPa${}^{-1}$).

It is nevertheless worth noting that all the above discussed pressure derivatives of the total magnetic moment are very small in their magnitude and their value can be possibly quite strongly influenced by other aspects of our calculations (performed for perfectly periodic static lattices without any phonon or magnon excitations). Moreover, the above discussed comparison of pressure derivatives is based on the assumption that our calculated results for individual states are valid for inclusions embedded into the Ni${}_{2}$MnSn. However, the surrounding matrix affects the inclusions by both strain-elastic field as well as a magnetic field. Moreover, the pressure derivative of the total magnetic moment can be altered by graduate flipping of individual Mn atoms from the FM-coupled to AFM-coupled state, or vice versa, and this mechanism is yet more difficult to include in our discussion. However, if a gradual flipping-related change would be from the lower-energy FM-coupled states into higher-energy AFM-coupled states with increasing pressure, the total magnetic moment would decrease even faster with pressure (and would be even more sensitive) in contrast to the fact that our experiments show Ni${}_{2}$MnSn to be less sensitive to the pressure. Therefore, some aspects related to the discrepancy between the theory and experiment in the case of Ni${}_{2}$MnSn will remain unexplained for the time being and will constitute a topic of future studies.

So, in general, our study shows that defects of various types can have a very strong influence on numerous properties of Ni${}_{2}$MnSn, in particular, both local and total magnetic moments and their pressure dependencies. The formation energies in Table 1 show that FM-coupled and AFM-coupled states are often energetically nearly degenerated when the formation energies differ by only a few meV per atom. In contrast to these very small energy differences, the AFM-coupled to FM-coupled state can have a very different magnetic properties.

## 5. Conclusions

We have performed an ab initio study of a series of stoichiometric Ni${}_{2}$MnSn states with austenitic (full Heusler type) structure using 16-atom computational supercells. In particular, we have focused on pressure-induced changes in their magnetic state as we have our own experimental data for austenitic Ni${}_{2}$MnSn [6]. Motivated by the facts that our calculations (i) give the total magnetic moment of the defect-free stoichiometric Ni${}_{2}$MnSn higher than the experimental value by 12.8% and (ii) predict it to be more sensitive to hydrostatic pressures, our study was focused on the role of point defects in this material. We have studied the effect of Mn-Ni, Mn-Sn and Ni-Sn swaps in the stoichiometric Ni${}_{2}$MnSn and we also compared states with both ferromagnetic (FM) and anti-ferromagnetic (AFM) coupling between (i) the swapped Mn atoms and (ii) the Mn atoms on the Mn sublattice for most of these atomic configurations (for some atomic configurations we were not able to stabilize them in our calculations). By analyzing the magnetic moments of states with swaps we show their complexity and significant influence on materials properties. In particular, they can lead to total magnetic moments twice smaller than those in the defect-free Ni${}_{2}$MnSn and pressure-induced changes in the total magnetic moment can be nearly three times larger but also smaller depending on the type of defects and the coupling of Mn atoms. Importantly, we find both qualitative and quantitative differences also in the pressure-induced changes of magnetic moments of individual atoms even for the same global magnetic state. Lastly, the FM-coupled and AFM-coupled states with very different magnetic properties have sometimes formation energies different only by a few meV per atom. It then seems that a few mechanisms acting at once contribute into the above mentioned theory-experiment discrepancy. The complexity of the Ni-Mn-Sn system make it on one hand quite challenging to study but, on the other hand, possibly allows for a flexible fine-tuning of functional properties of these materials within a theory-guided materials design based on utilizing properties of various types of defects.

## Figures and Tables

**Table 1:** Computed properties of the studied systems including the lattice parameters of the 16-atom supercells, formation energies $E_{f}$ (in eV per atom), total magnetic moments $\mu^{\mathrm{TOT}}$ (in $\mu_{B}$ per 4-atom formula unit, f.u.) and its pressure derivatives d(lnμ)/d *p*. States with a lower formation energy (from the pair of either FM-coupled or AFM-coupled one) have their formation energy printed in bold.

System	Mn-Mn Coupling	Lattice Parameter	$\mu^{\mathrm{TOT}}$	$E_{f}$	d(lnμ)/dp
		(Å)	($\mu_{B}$/f.u.)	(eV/atom)	(GPa${}^{-1}$)
Ni${}_{8}$Mn${}_{4}$Sn${}_{4}$ L2${}_{1}$ full Heusler	FM	6.059	4.09	−0.167	−0.0036
Ni${}_{8}$Mn${}_{4}$Sn${}_{4}$ Mn swaps Sn	FM	6.083	4.03	**−0.074**	−0.0035
Ni${}_{8}$Mn${}_{4}$Sn${}_{4}$ Mn swaps Sn	AFM	6.076	1.87	−0.066	−0.0047
Ni${}_{8}$Mn${}_{4}$Sn${}_{4}$ Mn swaps Ni	FM	6.058	3.75	**−0.113**	-0.0119
Ni${}_{8}$Mn${}_{4}$Sn${}_{4}$ Mn swaps Ni	AFM	6.053	2.09	−0.107	−0.0037
Ni${}_{8}$Mn${}_{4}$Sn${}_{4}$ 3 Mn swap 3 Ni	FM	6.054	3.58	−0.029	−0.0098
Ni${}_{8}$Mn${}_{4}$Sn${}_{4}$ 3 Mn swap 3 Ni	AFM	6.049	1.57	**−0.040**	−0.0107
Ni${}_{8}$Mn${}_{4}$Sn${}_{4}$ inverse Heusler	FM	6.048	3.51	−0.019	−0.0070
Ni${}_{8}$Mn${}_{4}$Sn${}_{4}$ Ni swaps Sn	FM	6.106	4.04	−0.012	−0.0048

## Data Availability

The data presented in this study are available on request from the corresponding author.
